# Inactivation of deposited bioaerosols on food contact surfaces with UV-C light emitting diode devices

**DOI:** 10.1128/aem.01093-24

**Published:** 2024-11-21

**Authors:** Aakash Sharma, Amritpal Singh, Brahmaiah Pendyala, Sampathkumar Balamurugan, Ankit Patras

**Affiliations:** 1 Department of Food and Animal Sciences, Tennessee State University 5717 , Nashville, Tennessee, USA; 2 Guelph Research and Development Centre, Agriculture and Agri-Food Canada Guelph 98662 , Guelph, Canada; Maisons-Alfort Laboratory for Food Safety, Maisons-Alfort, France

**Keywords:** food safety, bioaerosols, *Pseudomonas fragi*, UV-C LED, personal safety

## Abstract

The airborne transmission of infectious diseases and bioaerosol-induced cross-contamination pose significant challenges in the food, dairy, and pharma industries. This study evaluated the effectiveness of 279 nm UV-C LED irradiation for decontaminating bioaerosols, specifically containing microorganisms such as *Escherichia coli* (C3040- Kanamycin resistant), *Salmonella* Enteritidis (ATCC 4931), and *Pseudomonas fragi* (ATCC 4973), on food contact surfaces. Borosilicate glass, silicon rubber, and stainless steel (316L) surfaces were selected for experimentation for their usage in the food industry. A 50 µL cell suspension was aerosolized at 25 psi pressure using a 4-jet BLAM Nebulizer within a customized glass chamber and then deposited onto the surface of the coupons. The serial dilution approach was used for the microbial enumeration, followed by duplicate plating. With a low Root Mean Square Error (RMSE) and high *R*^2^ values, the biphasic kinetic model for UV-C inactivation curves of all three pathogens demonstrated the excellent goodness of fit parameters. At a UV-C dose of 6 mJ cm^−2^, glass surfaces showed the maximum microbial inactivation (i.e., 2.80, 3.81, and 3.56 log CFU/mL for *E. coli*, *Salmonella*, and *P. fragi*, respectively). Stainless steel and silicon rubber surfaces showed significant microbial inactivation, but log_10_ reductions observed were consistently lower than glass surface. Our research indicates that UV-C LEDs (279 nm) can effectively disinfect bioaerosols on food contact surfaces.

**IMPORTANCE:**

Food safety is a major public health concern, with contaminated food causing serious illnesses. UV-C light, used for germicidal action, is effective in disinfecting surfaces and is not subject to the same strict legal restrictions as chemical disinfectants, simplifying compliance with food safety regulations. In this study, we evaluated the efficacy of UV-C (279 nm) LED systems for inactivation of surface-deposited bioaerosols of kanamycin-resistant *Escherichia coli* (C3040), *Salmonella* Enteritidis (ATCC 4931), and *Pseudomonas fragi* (ATCC 4973). The research outcomes can be used to develop UV-based surface disinfection systems to minimize the risk of foodborne illnesses and enhance safety in high-traffic food preparation areas.

## INTRODUCTION

Food safety is a critical public health issue, as contaminated food can cause serious illnesses. Biological agents, such as bacteria, viruses, parasites, and fungi, can enter the food chain from production to consumption, posing significant health risks. For instance, *Salmonella* infections account for a significant number of foodborne pathogens affecting 48 million people annually (2018) in the United States alone (1, 2). The World Health Organization reports that foodborne diseases are responsible for approximately 420,000 deaths and 60 million illnesses annually worldwide (3). In 2018, an extensive *Escherichia coli* outbreak was traced back to romaine lettuce and caused harm to 210 individuals across 36 US states. Tragically, this outbreak resulted in 96 hospitalizations and 5 fatalities (4). Similarly, in 2019, an outbreak of *Salmonella* Newport linked to ground beef affected 403 people in 30 states, leading to 117 hospitalizations and 1 death (5). In addition to the pathogenic micro-organism, spoilage microflora contributes to food wastage. It is important to note that *Pseudomonas fragi* is one of the most prevalent pseudomonads responsible for meat spoilage (6, 7). Recognizing food safety and security as a collective responsibility is crucial in preventing and controlling foodborne illnesses.

Aerosols, tiny particles composed of liquid or gas, pose significant health and safety risks by spreading biological contaminants such as bacteria, viruses, and allergens (8). Bioaerosols are created when microorganisms are trapped inside air or water bubbles, ranging in size from less than 1 to over 100 µm (9). The extent of aerosol deposition is influenced by several factors, including the particle size of the aerosol, airflow patterns within the environment, surface properties, and humidity levels. Smaller particles, for example, tend to remain airborne longer and, thus, have a higher likelihood of deposition over a wider area (10). A study explored the dynamics of aerosol deposition in various environmental conditions highlighting the importance of controlling aerosol production and mitigating their impact in food processing and preparation areas to ensure food safety (11). When bioaerosols are deposited, they can act as nuclei for biofilm formation on food contact surfaces, which can lead to contamination of food products (12). Recent studies have observed the complexities of disinfecting surfaces using ultraviolet (UV-C) light in food-processing environments (13). In various scientific studies on surface disinfection, UV-C microbial inactivation rates (*k*) tend to follow a decreasing order, PVC < stainless steel < Teflon < silicon (14–16).

UV-C light, known for germicidal action, is utilized in a variety of applications, such as water treatment, air purification, and surface disinfection (17–19). UV-C disinfection devices are available in various design formats, ranging from portable units designed for small areas to larger systems suitable for entire rooms. UV-C light functions by damaging the DNA and/or RNA of microorganisms by inducing cross-linking between thymine and cytosine, leading to the formation of dimers mainly cyclobutyl pyrimidine dimers (CPD) and pyrimidine (6-4) pyrimidone dimers (6-4PPs) that interrupt DNA processes (20, 21), thereby inhibiting their essential functions and replication. Research has identified that the most effective wavelength for UV-C disinfection is between 254 and 275 nm (22). A recent study reported, at 279 nm, the microbes exhibited protein damage along with DNA damage (23). Surfaces that are heavily contaminated may require a higher dose of UV-C light (24). Low-pressure mercury lamps have been the primary source of UV-C light, but the Minamata Convention on Mercury in January 2020 aimed at reducing mercury use due to health risks (14). Advances in material science have led to the development of high-intensity UV-C LEDs, a previously unfeasible option due to poor semiconductor quality (25). UV-C technology is generally not subject to the same stringent legal restrictions that govern the use of many chemical disinfectants. This aspect can simplify compliance with food safety regulations and reduce the administrative burden associated with using certain chemicals in food-processing environments (26).

Limited studies have been conducted with UV-C LEDs on food contact surfaces. A study found that UV-LEDs in the 259–275 nm range can be as or more effective than traditional lamps at 253.7 nm in reducing foodborne pathogens (27). Another study also demonstrated that 266 nm UVC-LEDs achieved over 5 log reductions in *E. coli* O157:H7 and S. Typhimurium, significantly more effective than traditional UV lamps at the same dosages to disinfect food contact surfaces (28). This study aims to evaluate the effectiveness of 279 nm UV-C LED irradiation as a non-thermal method for decontaminating food contact surfaces deposited with bioaerosols, specifically containing microorganisms such as *E. coli*, *Salmonella* Enteritidis, and *P. fragi*, on food contact surfaces. A secondary objective is to develop kinetic models and understand the effect of surface properties on microbial rate constants.

## MATERIALS AND METHODS

### Bacterial strains and culture conditions

*E. coli* C3040 (kanamycin resistant strain) was obtained in freeze-dried form from New England Biolabs (Ipswich, Massachusetts, USA). *Salmonella* Enteritidis ATCC 4931 and *P. fragi* ATCC 4973 were obtained from the American Type Culture Collection (Manassas, Virginia) in freeze-dried form. Tryptic Soy Broth (211825, Becton Dickinson and Company, Le Pont de Claix, France) was used to propagate all microorganisms. For propagation, 2% inoculation (100 µL in 5 mL and 1 mL in 50 mL) from active culture was transferred using a pipette to tryptic soy broth followed by incubation at an optimum growth time-temperature combination for the microorganisms. Both *E. coli* and *Salmonella* were incubated at 37°C for 18 h; however, *P. fragi* was incubated at 30°C for 24 h. For plating, Tryptic Soy Agar (236950, Becton Dickinson and Company, Le Pont de Claix, France) was used for incubation at optimum time-temperature combination.

### Sample preparation

To prepare the sample for aerosolization tests, the microbial cells were harvested from 5 mL of fresh and active culture utilizing centrifugation at 5,000 × *g* for 5 min in stationary phase. The obtained cell pellets were washed twice using 0.1% (wt/vol) phosphate buffer saline (PBS, Becton Dickinson, New Jersey, USA), followed by re-suspension in sterile deionized (DI) water. During the re-suspension process, the volume of DI water used was one-tenth of the original culture volume, thereby concentrating the cells 10-fold in the resulting suspension. After concentration, the suspension was used for aerosol generation and testing. After aerosolization in each experiment (including replicates), the coupon surface had an average microorganism concentration of 6.1 log CFU cm^−2^ during aerosolization.

### Aerosolization

**Fig 1:**
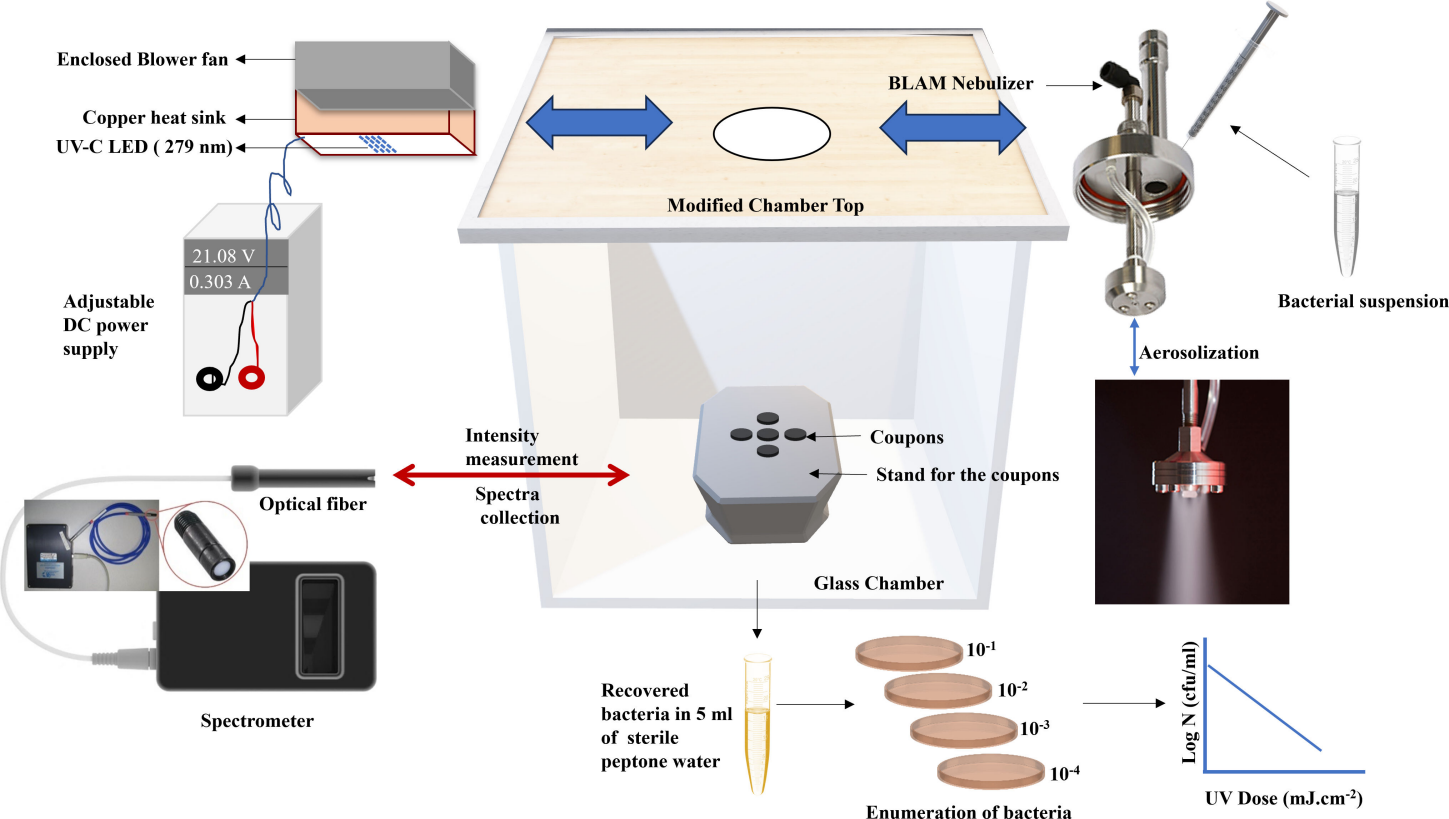
Graphical abstract.

**Fig 2:**
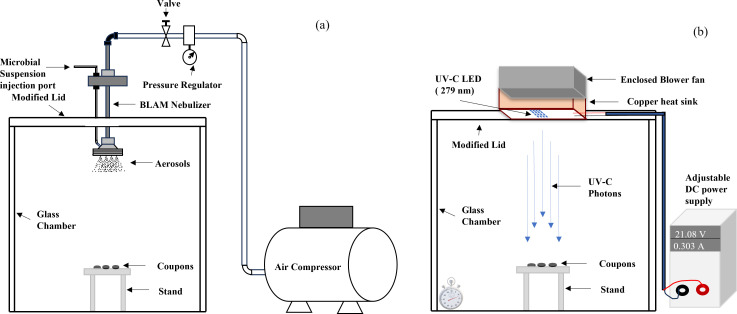
Aerosolization setup (a) and UV-C treatment setup (b).

Stainless steel (316L), silicone rubber, and borosilicate glass coupons, each with a diameter of 0.5 inches (12.7 mm), were obtained from BioSurface Technologies Corporation, Bozeman, Montana, USA. These coupons were thoroughly washed with surfactant (5% Tween 80 in DI Water) and then sanitized by immersing in 70% ethanol for 10 min followed by pat drying using sterile paper towels. After sanitation, the coupons were positioned on a sanitized jack (surface sanitized with 70% ethanol) as depicted in Fig. 1 and 2a. The microbial inactivation testing was conducted in a modified glass chamber measuring 11 × 11 × 11 inches, equipped with a circular cutout opening at the top (Fig. 2a). This chamber was placed within a Biosafety Level 2 (BSL-2) (with controlled exhaust through HEPA filters) safety cabinet throughout the experiment. For bioaerosol generation, a 4 jet Blaustein Atomizing Module (BLAM) type atomizer (CH Technologies, Westwood, New Jersey, USA) was employed. Fifty microliters of a test suspension was injected at a rate of 300 µL min^−1^ into the nebulizer using a syringe, aided by compressed air at 25 psi. The bioaerosols, thus, generated settled on the coupon surfaces, aligned directly below the nebulizer outlet. Upon completion of the aerosolization process, the nebulizer was removed, and a UV-C LED module was installed in the pre-marked slots (Fig. 2b). To prevent cross-contamination, the chamber was sterilized by spraying 70% ethanol in between the experiments.

### UV exposure

**Fig 3:**
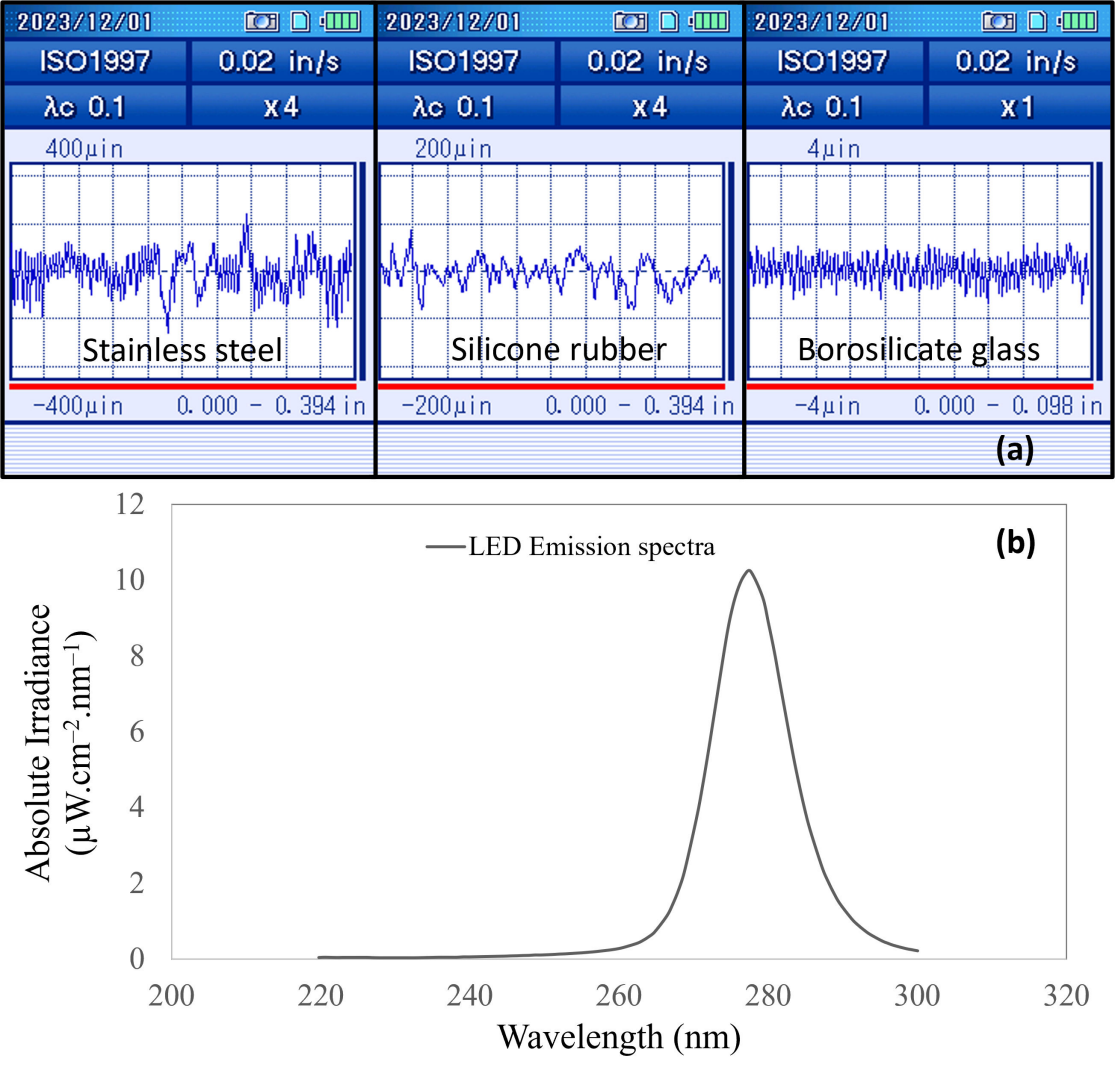
A graphical representation of the protrusions and crevices of the SS (stainless steel), SR (silicone rubber), and BG (borosilicate glass) surfaces (a); UV-C LED emission spectra (b).

A panel comprising 16 UV-C Light Emitting Diodes (LEDs), provided by IRTRONIX, Torrance, CA, USA, was employed for the bioaerosol inactivation study as shown in Fig. 2b. These LEDs, attached to a heat sink, emitted a peak wavelength of 279 nm (±12 nm). The LEDs’ emission spectra, broader than those of low-pressure lamps, are illustrated in Fig. 3b. The LED module was powered using a 180W, 60 V-8A DC Multirange Power Supply from BK Precision, Yorba Linda, California. To measure incident surface irradiance, we utilized a high-sensitivity spectrophotometer (QE Pro series, Ocean Optics, Dunedin, FL, USA) which measured absolute spectral irradiance (µW cm^−^² nm^−1^). The spectrophotometer was connected to a solarization-resistant optical fiber with a Spectralon cosine corrector, designed for a 180° field of view. Spectralon, being a Lambertian diffuse material, exhibits more than 95% reflectance in the 250–400 nm range. We assessed the consistency and spectral shift of the LED system and experimental setup within the exposure area using the same high-sensitivity spectrophotometer. The findings confirmed that the exposure area for the coupons maintained stable and consistent irradiance. Surface irradiance, or intensity, was calculated by summing the irradiance over the relevant wavelength range. The total UV dose delivered (mJ cm^−^²) was determined by multiplying the UV intensity (mW cm^−^²) by the exposure time (seconds). A range of UV-C exposures, from 0 to 6 mJ cm^−^², was delivered to the test microorganisms. All microbial experiments were performed in triplicates.

### Recovery and enumeration after UV-C treatments

Following the UV-C treatment, the coupons were aseptically transferred using sterile tongs into 15 mL centrifuge tubes, each containing 5 mL of 0.1% peptone water (Bio-Rad Laboratories, Inc., Hercules, CA, USA), and the tubes were then vortexed at high speed for 2 min to recover UV-C treated bacteria into suspension. For the enumeration of surviving microorganisms, appropriate serial dilutions (in 0.1% peptone water) were spread-plated onto tryptic soy agar (TSA) plates. These plates were incubated at the specific optimum growth time-temperature combination for each microorganism under study. Colony counts were performed on TSA plates that displayed isolated colonies in the range of 25–250 with a detection limit of 1.4 Log_10_ CFU mL^−1^. The results were then expressed as colony-forming units per milliliter of the sample (CFU mL^−1^). The data were further converted to CFU cm^−2^ using the surface area of the coupon (1.266 cm^2^) and the volume used for microbial recovery (5 mL). The microbial plating was performed in duplicate for each sample, and the entire experiment was conducted in triplicate to reduce experimental error.

### Surface roughness measurements

A portable surface roughness tester SJ-210 from Mitutoyo (Sakado, Takatsu-Ku, Kawasaki, Kanagawa, Japan) was utilized for the determination of surface roughness values for stainless steel, silicon rubber, and borosilicate glass coupons. The SJ-210 V.1.210 version software with the standard configuration was used. The measurement conditions (Lc—0.1 in, Ls—320 μin, sampling lengths (N)—4, prelength—OFF, pitch—59.1 μin) were kept identical for all three coupon types with 0.02 in s^−1^ probe (sensor) speed. Each coupon was tested for surface roughness, and the average roughness (µm) from each coupon was an average of four sampling lengths.

### Statistical analysis and mathematical modeling

All experiments were performed in triplicate, and surviving bacterial populations following UV-C treatment are presented as mean Log CFU cm^−2^ in Table 1. To assess the effects of UV-C, one-way ANOVA with Tukey’s HSD test was conducted on microbial log reduction and dose delivered using R-studio. The data were presented as means ± standard deviation, and statistical significance was tested at a 5% significance level. All log reductions from the UV-C inactivation treatments were recorded, and models were fitted using Microsoft Excel and the GInaFit Tool (Excel Add-in). The model fit statistics, including adjusted *R*^2^ and RMSE, were compared among the competing models.

**TABLE 1:** Microbial log reduction per unit area for deposited bioaerosol inactivation using UV-C LEDs^*a*^

Microorganism	Dose (mJ cm^−2^)	Log reduction (Log_10_ cm^−2^)					
		Stainless steel		Silicon rubber		Borosilicate glass	
		Mean	Std. Dev.	Mean	Std. Dev.	Mean	Std. Dev.
*E. coli* (C3040)	0	^a^0.00	0.00	^a^0.00	0.00	^a^0.00	0.00
	1	^b^1.70	0.14	^b^1.66	0.09	^b^2.18	0.10
	2	^b^1.90	0.04	^c^2.01	0.11	^c^2.42	0.08
	4	^c^2.29	0.05	^d^2.28	0.05	^d^2.89	0.20
	6	^d^2.63	0.10	^e^2.50	0.02	^e^3.39	0.06
*Salmonella* Enteritidis (ATCC 4931)	0	^a^0.00	0.00	^a^0.00	0.00	^a^0.00	0.00
	1	^b^2.10	0.03	^b^2.25	0.06	^b^3.09	0.03
	2	^b^2.41	0.01	^b^2.43	0.07	^c^3.39	0.01
	4	^c^3.32	0.03	^c^3.27	0.04	^d^3.90	0.01
	6	^c^3.63	0.05	^c^3.51	0.08	^e^4.40	0.02
*P. fragi* (ATCC 4973)	0	^a^0.00	0.00	^a^0.00	0.00	^a^0.00	0.00
	1	^b^2.13	0.23	^b^2.41	0.19	^b^2.92	0.06
	2	^c^2.57	0.13	^c^3.02	0.14	^c^3.34	0.05
	4	^d^3.08	0.19	^d^3.33	0.12	^d^3.57	0.05
	6	^e^3.74	0.23	^e^3.67	0.25	^e^4.16	0.03

^*a*^ Different letters within rows for specific microorganisms and surface type represent a statistically significant difference between mean inactivation with *P* < 0.05.

A biphasic (non-linear) model, within GInaFiT software, accurately depicted the kinetics of microorganism inactivation by UV-C for current study. The selected model has a good fit to data and goodness of fit parameters such as *R*^2^, root mean square error (RMSE), and rate constants. Also, the software can determine the efficacy of the selected models in fitting the data. The equation for the biphasic model is


(1) $$N=N_{0}\times e^{-(k_{1}\times t)}+N_{R}\times e^{-(k_{2}\times t)}$$

where *N* is the number of surviving microorganisms at time *t*, *N*_0_ is the initial number of microorganisms, *k*_1_ is the inactivation rate constant for the first phase, *k*_2_ is the inactivation rate constant for the second phase, and *N*_*R*_ is the residual number of microorganisms. Microbial rate constant refers to the numerical value that describes the rate at which a microbial population changes under specific conditions. This model can account for the non-linear nature of the inactivation curve and the presence of resistant microorganisms that survive the initial phase of the treatment. Often, the equation is expressed in a logarithmic form to linearize the biphasic nature:


(2) $$\log_{10}\left(N\right)=\log_{10}\left(N_{0}\right)+\log_{10}\left(f\times e^{{(-k}_{1}\times t)}+N_{R}\times e^{{(-k}_{2}\times t)}\right)$$

For special cases, if *N*_*R*_ = 0 or *N*_*R*_ = 1, the model reduces to a single-phase first-order kinetic model. The model can be modified or expanded based on specific experimental conditions or observed microbial inactivation patterns.

## RESULTS AND DISCUSSION

### UV-C LEDs absolute irradiance, emission spectra, and UV-C dose

In Fig. 3b, the UV-C LED emission spectra are displayed, showing a broader range compared to low-pressure mercury lamps. Low-pressure mercury lamps have been extensively studied and are presently employed due to their narrow emission, 90% output at the peak wavelength (254 nm) with a 4 nm spread on either side. In contrast, the UV-C LED used in this study exhibited a broader (>5%) emission spread of 12 nm on both sides from its peak wavelength (279 ± 12 nm). In the experimental settings, the average fluence rate or irradiance on the coupon surface was determined to be 0.070 ± 0.002 mW cm^−2^ from the optical source. To attain the desired power levels, the UV-C LED was consistently operated at 0.303 A and 21.08 V of current and voltage.

### Surface roughness

Surface roughness values of different food contact surfaces used in the present study are presented in Table 2. The average deviation from the centerline within the sample area is Ra. Rq (also known as Root mean square deviation) is the root mean square of deviations within the sampling area. Rz represents the average of sequential peak to valley within the sampling length. Borosilicate glass had the lowest surface roughness attributes among all three surfaces. Followed by silicone rubber and the highest surface roughness was for the stainless-steel coupons.

**TABLE 2:** Surface roughness data (*N* = 5) for food contact surfaces

Surface type	Surface roughness attributes (µm)		
	Ra^*a*^	Rq^*b*^	Rz^*c*^
Borosilicate glass	0.020 ± 0.009	0.027 ± 0.013	0.169 ± 0.108
Silicone rubber	0.576 ± 0.030	0.728 ± 0.061	4.006 ± 1.030
Stainless steel (316 L)	1.473 ± 0.091	1.854 ± 0.116	9.437 ± 0.926

^*a*^ Ra is the average deviation from the centerline within the sample area.

^*b*^ Rq (also known as root mean square deviation) is the root mean square of deviations within the sampling area.

^*c*^ Rz is the average of sequential peak to valley within sampling length.

Kim and Kang (14) reported the Ra values of 0.0204 ± 0005 µm, 0.96 ± 0.02 µm, and 0.58 ± 0.07 µm for glass, silicone, and stainless steel No. 4, respectively. A recent study observed the surface roughness value of 0.48 µm for glass (29). The silicone rubber properties are usually determined by the curing process, and the technique used determines the polymer properties (30). Another study also reported the Ra values ranging from 0.11 to 1.32 µm for various stainless-steel surfaces after surface treatments (31). However, it is important to note that the surface roughness of stainless steel can vary substantially depending on its grade. The numerous grades of stainless steel possess different roughness values, which may explain the deviation in results. Additionally, we observed that Rz, which represents the average of sequential protrusions to crevices, was highest for stainless steel and silicone rubber. This observation suggests the potential existence of hiding spots for microorganisms within the crevices, which could have implications for food safety and hygiene.

### UV-C microbial inactivation

#### E. coli

**Fig 4:**
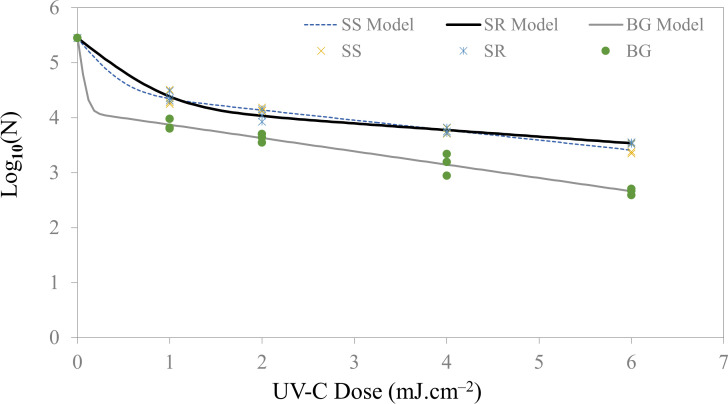
Microbial inactivation curve (survival colonies vs UV Dose) for *E. coli* C03040 bioaerosols deposited on SS (stainless steel), SR (silicone rubber), and BG (borosilicate glass) coupons using a UV-C light-emitting diodes.

In this study, we delivered a total of 5 UV-C doses for each type of food contact surface. Specifically, we investigated the UV-C inactivation of kanamycin-resistant *E. coli* C3040 using UV-C LED with a peak wavelength of 279 nm. Figure 4 illustrates the survival curve of *E. coli*. These survival curves conformed to the biphasic model, as indicated by the high *R*^2^ values and low RMSE values for all three surface types, as detailed in Table 3. It also provides the rate constants (*k*_max1_, *k*_max2_) values for each of the surfaces. We observed a substantial reduction in microbial load, with a maximum reduction of 2.8 Log_10_ CFU mL^−1^ achieved with the highest dose of 6 mJ cm^−2^. However, this microbial inactivation was primarily evident in the case of borosilicate glass. Although UV-C LED inactivation of *E. coli* on stainless steel and silicone rubber was lower than that observed on borosilicate glass, *E. coli* inactivation on stainless steel was not significantly different (*P*-value > 0.05) compared to inactivation on silicone rubber at 6 mJ cm^−2^. Specifically, the microbial log reduction on stainless steel and silicone rubber was 2.03 ± 0.10 and 1.91 ± 0.02 log CFU mL^−1^, respectively, at the highest dose delivered (6 mJ cm^−2^) compared to 2.8 log CFU mL^−1^ on borosilicate glass. At a lower dose of 1 mJ cm^−2^, a minimum of 1 log inactivation was observed across all surface types. Notably, microbial inactivation was consistently higher on glass surfaces, regardless of the dosage level, with approximately 0.5 log higher reduction compared to the other stainless steel and silicone rubber. The similar log inactivation for stainless-steel coupons in comparison to silicone rubber may be due to the other surface properties mainly reflectivity and diffused reflectance despite having relatively higher surface roughness (deeper crevices and higher protrusions). Due to the diffused reflectance, the photons can divert toward the crevices of the stainless steel once reflected from the edges.

**TABLE 3:** Kinetic constants and goodness of fit parameters for bi-phasic model

	Surface	*k*_max1_ ^*a*^	*k*_max2_ ^*b*^	*R* ^2*c*^	*R*^2^ adj^*d*^	RMSE^*e*^
*E. coli* (C3040)	Stainless steel	5.25 ± 0.70	0.42 ± 0.01	0.991	0.988	0.0781
	Silicon rubber	3.21 ± 0.07	0.28 ± 0.01	0.993	0.991	0.067
	Borosilicate glass	28.48 ± 3.59	0.56 ± 0.01	0.991	0.990	0.103
*Salmonella* Enteritidis (ATCC 4931)	Stainless steel	4.95 ± 0.48	0.70 ± 0.03	0.972	0.964	0.211
	Silicon rubber	8.14 ± 0.84	0.62 ± 0.02	0.972	0.965	0.201
	Borosilicate glass	6.96 ± 0.12	0.57 ± 0.01	0.999	0.998	0.058
*P. fragi* (ATCC 4973)	Stainless steel	4.87 ± 0.08	0.67 ± 0.01	0.999	0.998	0.044
	Silicon rubber	4.60 ± 0.04	0.37 ± 0.01	0.998	0.997	0.056
	Borosilicate glass	6.50 ± 0.12	0.47 ± 0.01	0.997	0.996	0.079

^*a*^ *k*_max1_ is the inactivation rate constant at the start of inactivation phage.

^*b*^ *k*_max2_ is the inactivation rate constant after tailing started.

^*c*^ *R*^2^ represents variance proportion which is explained by an independent variable for a dependent variable.

^*d*^ Adjusted *R*^2^ indicates how well data points fit a curve in accordance with the model.

^*e*^ Root mean sum of squared error.

In this study, the microbial inactivation increased in a dose dependent manner for UV-C dose range of 1–6 mJ cm^−2^. The biphasic model utilized in this study exhibited an initial high log inactivation rate, followed by a gradual decline in the rate constant. This phenomenon can be attributed to the stacking of bacteria on surfaces, forming multiple layers that shield the bottom layers from UV photons, thereby protecting some microbes from irradiation. Guo and Chen (29) reported disinfection efficiency for *E. coli* ATCC 15597 on various surface materials and measured their *Z*-values. Among non-porous surfaces, copper exhibited the highest disinfection efficiency due to its inherent antimicrobial properties, followed by glass, which had a *Z*-value of 0.0443 ± 0.0166 m^2^ J^−1^. Additionally, Kim and Kang (32) observed a 1 log_10_ reduction of *E. coli* O157:H7 at a UV-C dose of 0.8 mJ cm^−2^. The research was focused on bioaerosol inactivation in chamber-type air disinfection system using UV-C LEDs, with the survival kinetics following the Weibull model, till the highest dose of 1.5 mJ cm^−2^. Another study by Kim and Kang (14) demonstrated a 1.2 log reduction when delivering 2 mJ cm^−2^ for stainless steel coupons using 279 nm LED although they employed the liquid droplet method instead of bioaerosol deposition. Pendyala et al. (33) also reported 2.90 ± 0.12 mJ cm^−2^ as the *D*_10_ value of *E. coli* ATCC 25922 on the stainless steel surface using the liquid loading method. The liquid loading method has two major limitations. The first one being overlapping (multi-layered) microorganisms in droplets which results in non-uniform dose delivery. Second, the drying process will contribute to some microbial inactivation which can be corrected by having appropriate controls, but it may change or alter the microorganism’s sensitivity. Despite the differences, the *E. coli* inactivation number is close to what we observed in our study, i.e., 1.30 log_10_ reduction.

Furthermore, various studies have assessed the efficacy of UV-C treatments in the inactivation of antibiotic-resistant bacteria, consistent with our findings regarding kanamycin-resistant *E. coli* (34–36). Numerous studies have demonstrated *E. coli* in suspension exhibiting the linear trend having a generalized *D*_10_ value of 2–4.5 mJ cm^−2^ (37–42). In comparison to this study, microbial inactivation of bioaerosols deposited on surfaces requires a lower average dose for similar log inactivation. Another study demonstrated a dose of 2.70 mJ cm^−2^ is required for 1 log inactivation of *E. coli* ATCC 25922 on the stainless steel surface as a droplet (15). However, for bioaerosol inactivation of *E. coli* C3040 on stainless steel, our study demonstrated that only a 1 mJ cm^−2^ UV-C dose was required using the same LED system.

#### *Salmonella* Enteritidis

**Fig 5:**
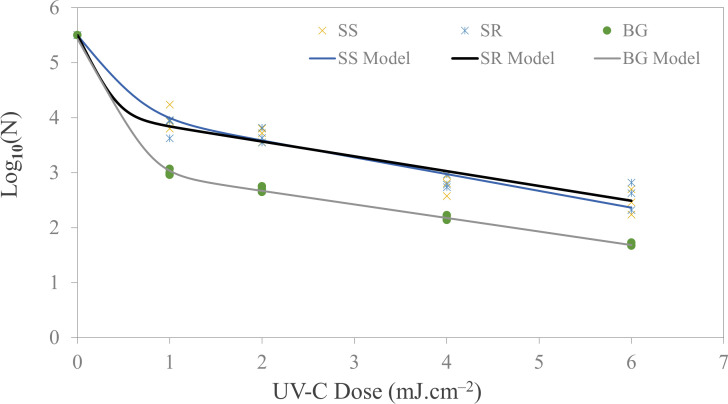
Microbial inactivation curve (survival colonies vs UV Dose) for *Salmonella* Enteritidis (ATCC 4931) bioaerosols deposited on SS (stainless steel), SR (silicone rubber), and BG (borosilicate glass) coupon using UV-C (279 nm wavelength) LEDs.

Figure 5 presents the *Salmonella* survival curve for all three surfaces, illustrating the relationship between UV-C dose and log survival. Across all surface types, the microbial survival kinetics exhibited a consistent biphasic model, characterized by an *R*^2^ value exceeding 0.96 and low RMSE values. Notably, when the maximum dose of 6 mJ cm^−2^ was applied to the borosilicate glass coupon, we achieved 3.81 log reduction. Similar to our observations with *E. coli*, the highest log inactivation was consistently observed on glass coupons, followed by stainless steel, with silicone rubber exhibiting the lowest inactivation. For stainless steel and rubber, we noted reductions of 3.03 logs and 2.91 logs CFU mL^−1^, respectively, at the highest dose delivered. It is noteworthy that while an increase in UV-C dose led to an increase in microbial inactivation, the inactivation rate constant (*K*_max2_ < *K*_max1_) dropped considerably at higher doses. Table 3 presents the *k*_max_ values ranging from 0.57 to 8.14 for the fitted biphasic model having at least 0.96 *R*^2^ and a maximum RMSE value of 0.211 for *Salmonella* inactivation on food contact surfaces.

Furthermore, in line with our observations, we can draw conclusions from prior research. A similar non-linear inactivation model for *Salmonella* spp. was reported on food contact surfaces (43). The study, involving a *Salmonella enterica* cocktail on various surface types and finishes, also followed a biphasic model. It exhibited a higher rate of inactivation (rate constant*—K*_max1_) for lower doses followed by a substantially reduced rate of inactivation (rate constant—*K*_max2_) for higher doses. The results demonstrated that 7–13 s of UV-C exposure resulted in approximately 3 log_10_ reduction for a mixed strain cocktail of *Salmonella*, equivalent to 4–7 mJ cm^−2^ UV-C dose. Similarly, Lim and Harrison (44) reported a 2.75 log_10_ reduction in the *Salmonella* cocktail population with an exposure of 5 s (3.3 mJ cm^−2^). Another study found similar inactivation kinetics (16). They achieved rapid 1.3 log_10_ inactivation with 15 s of exposure for the *Salmonella* cocktail consisting of multiple strains. However, they observed that as the UV-exposure increased, the microbial inactivation rate dropped considerably.

Collectively, these findings emphasize the complexity of UV-C inactivation kinetics for *Salmonella* on various surfaces suggesting the importance of dosage and surface type in achieving effective microbial reduction. It is clear that the biphasic model is a common trend demonstrated in multiple studies (43, 44), with an initial rapid inactivation phase followed by a slower phase at higher doses. Understanding these dynamics will be vital for optimizing UV-C treatment protocols for food safety applications.

#### Pseudomonas fragi

**Fig 6:**
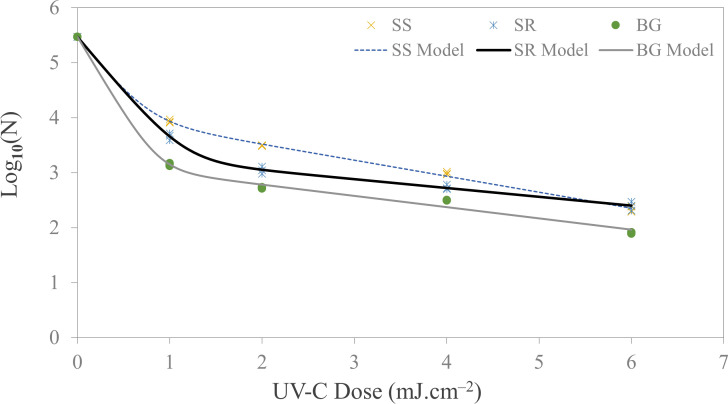
Microbial inactivation curve (survival colonies vs UV dose) for *P. fragi* (ATCC 4973) bioaerosols deposited on SS (stainless steel), SR (silicone rubber), and BG (borosilicate glass) coupon using UV-C (279 nm wavelength) light-emitting diodes.

This study also investigated the impact of UV-C irradiation on the population of *P. fragi* across three different surfaces: borosilicate glass, stainless steel, and silicon rubber. The highest UV dose applied, at 6 mJ cm^−2^, resulted in a significant (*P* value < 0.05) reduction of *P. fragi* population by 3.56 log_10_ on borosilicate glass coupons. Notably, borosilicate glass demonstrated the highest level of inactivation compared to the other surfaces. UV-C treatment on stainless steel and silicon rubber coupons achieved reductions of 3.14 log_10_ and 3.08 log_10_ CFU mL^-1^, respectively. The log inactivation observed for *P. fragi* under UV-C exposure also followed a non-linear trend (Bi-phasic) similar to that observed with *E. coli* and *Salmonella*. Figure 6 illustrates the survival curve for *P. fragi* as a function of the delivered dose. The model fitting for all surfaces exhibited an *R*^2^ value exceeding 0.99 and an RMSE value less than 0.079, indicating excellent model fitting.

Remarkably, even at a modest UV dose of 1 mJ cm^−2^, all surfaces experienced a reduction of slightly over 1.5 log_10_CFU in *P. fragi* population. However, as the UV dose increased beyond 1 mJ cm^−2^, the rate of microbial inactivation represented by inactivation rate constant (*K*_max1_ > *K*_max2_) did not increase proportionally; in contrast, it decreased substantially. Figure 6; Table 3 clearly demonstrate that glass consistently exhibited the highest microbial inactivation regardless of the dose delivered. 1.54 to 3.56 log_10_ inactivation of *P. fragi* deposited bioaerosols was achieved when exposed to UV-C doses ranging from 1 to 6 mJ cm^−2^ across the three different surfaces. Given the limited existing research on *P. fragi*, particularly regarding UV-C inactivation on surfaces, we referred to literature data to closely related species. A study reported UV-C inactivation of *Pseudomonas fluorescens* using 275 and 285 nm LEDs on stainless steel surfaces (45). They found *D*_90_ values of 2.3 and 1.5 mJ cm^−2^ for 275 and 285 nm UV-C LEDs, respectively, achieving a 4 log_10_ reduction at a 6 mJ cm^−2^ UV-C dose using 285 nm LEDs.

For all microbes tested, the stainless-steel coupons had higher microbial inactivation compared to silicone rubber due to differences in surface properties, namely, reflectivity and diffused reflectance. The microbial inactivation increased with higher UV-C doses but followed the biphasic model, which showed an initial high rate of microbial inactivation, followed by a gradual decline, possibly due to bacteria forming layers that shield lower layers from UV-C. Additionally, surface roughness may also play a role in microbial survival during UV-C treatment. Figure 3a illustrates the protrusions and crevices on coupon surfaces at baseline. Higher inactivation of bioaerosols on glass coupons may be attributed to their low Ra and Rq values. In contrast, stainless steel, and silicone rubber surfaces, with their protrusions, crevices (Fig. 3) and high roughness attributes, offer hiding spots for bioaerosols, resulting in a higher survival rate compared to glass coupons. While some experts argue that smoother surfaces are more hygienic and conducive to microbial inactivation (46–48), others suggest that the connection between roughness and microorganism adhesion or removal remains uncertain (31, 49, 50). Guo and Chen (29) conducted a study using sandpapers with different roughness levels and found no significant difference in *E. coli* inactivation between different roughness levels although the lower range of roughness (0.1–10 µm) was not studied. According to the current investigation and literature evaluation, surface roughness may influence microbial inactivation among other material properties (hydrophobicity, reflectivity, diffused reflectance), but it cannot be generalized.

### Conclusions

The utilization of UV-C LEDs, operating at a wavelength of 279 nm, demonstrated its effectiveness in inactivation of *E. coli* (C3040- Kanamycin resistant), *Salmonella* Enteritidis (ATCC 4931), and *P. fragi* (ATCC 4973) settled bioaerosols on food contact surfaces. The microbial inactivation ranged from 1.7 to 4.4 Log_10_CFU.cm^−2^ for all three surfaces varied due to the dose delivered and the microorganism treated. Microbial inactivation is influenced by a combination of factors, including the specific type of microorganism present and the inherent properties of the surface being treated. Our results could potentially be leveraged to develop innovative UV-based surface disinfection systems which may contribute to safer environments, reduce health risks, and can alter surface disinfection practices in the future.

## Data Availability

The data used to support the findings are included in the article. Further raw data can be provided on request to the corresponding authors.
